# Remnant cholesterol and all-cause mortality risk: findings from the National Health and Nutrition Examination Survey, 2003-2015

**DOI:** 10.3389/fendo.2024.1417228

**Published:** 2024-07-19

**Authors:** Muhan Bai, Jiangquan Liao, Yan Wang, Mengqi Liang, Chuan Wang, Jie Zhang, Mingjing Shao

**Affiliations:** ^1^ Beijing Anzhen Hospital, Capital Medical University, Beijing, China; ^2^ National Integrated Traditional and Western Medicine Center for Cardiovascular Disease, China-Japan Friendship Hospital, Beijing, China; ^3^ Affiliated Hospital 3, Beijing University of Chinese Medicine, Beijing, China; ^4^ Department of Cardiac Surgery, Beijing Anzhen Hospital, Capital Medical University, Beijing, China

**Keywords:** remnant cholesterol, all-cause mortality, cardiovascular disease, cause-specific mortality, NHANES

## Abstract

**Aims:**

Cholesterol carried in triglyceride-rich lipoproteins, also called remnant cholesterol, is increasingly acknowledged as an important causal risk factor for atherosclerosis. Elevated remnant cholesterol, marked by elevated plasma triglycerides, is associated causally with an increased risk of atherosclerotic cardiovascular disease. However, the association with all-cause mortality and cause-specific mortality is inconclusive. This study aimed to test the hypothesis that remnant cholesterol levels and plasma triglycerides are associated with increased all-cause mortality and mortality from cardiovascular disease, cancer, and other causes.

**Methods and results:**

Using a contemporary population-based cohort, 7,962 individuals from the National Health and Nutrition Examination Survey (NHANES) aged over 40 years at baseline in 2003–2015 were included. During up to 109.2 (± 1.44) months of follow-up, 1,323 individuals died: 385 individuals died from cardiovascular disease, 290 from cancer, 80 from cerebrovascular disease, and 568 from other causes. Compared with the middle tertile remnant cholesterol level, multivariable-adjusted mortality hazard ratios were 1.20 (95% confidence interval 1.02–1.40) for all-cause mortality. For the highest tertile remnant cholesterol level, multivariable-adjusted mortality hazard ratios were 1.21 (95% confidence interval 1.05,1.40). Our conclusions remained stable in subgroup analyses. Exploratory analysis of the cause of death subcategories showed corresponding hazard ratios of 1.25 (1.13–1.38) for Non-cardiovascular and Non-cerebrovascular Death for lower remnant cholesterol individuals, 1.47 (1.01–2.15) for cancer death for lower remnant cholesterol (RC) individuals, and 1.80 (1.36–2.38) for cancer death for higher RC individuals.

**Conclusion:**

RC levels were associated with U-shaped all-cause mortality. RC was associated with mortality from non-cardiovascular, non-cerebrovascular, and cancer, but not from cardiovascular causes. This novel finding should be confirmed in other cohorts.

## 1 Introduction

Remnant cholesterol (RC), a marker of cholesterol that remains undetected by standard lipid assays, has recently received increasing attention as a potential risk factor for CVDs (1). RC refers to the cholesterol contained in RLP particles, the cholesterol in the VLDL and IDL in the fasting state, and the cholesterol in the CM remnants in the postprandial state (2). The calculated RC (CRC) was defined as total cholesterol (TC) minus LDL-C minus HDL-C (3), which did not require special detection equipment and was more convenient and more accessible to implement in clinical diagnosis and therapy.

Studies have suggested a strong association between elevated RC levels and an increased risk of CVD events (4–6). Previous studies reveal that RC is associated with cardiovascular disease, inflammation, diabetes, frailty, and metabolism syndrome (7–10), but there are few studies on RC and all-cause mortality. In addition, RC is also associated with frailty, metabolic syndrome, and hepatic steatosis in middle-aged and elderly individuals (9–11). A cohort study involving 90,000 people showed that RC levels were associated stepwise with all-cause mortality. The FAVORIT study associated higher RC levels with higher all-cause mortality. A study has shown that the mortality rate of heart failure patients decreases as the level of RC increases. A study has shown that the mortality rate of heart failure patients decreases as the level of RC increases.

Our study aimed to determine whether RC is connected to mortality and causes of death in the middle-aged and elderly individuals by exploring the relationship between RC and all-cause mortality and specific causes of death in the middle-aged and elderly individuals.

## 2 Materials and methods

### 2.1 Study population

The NHANES, applying a stratified, multistage probability sample of non-institutionalized civilians, is a cross-sectional investigation to obtain basic information and healthy conditions. The study design and data collection methods have been previously documented (12). The National Center for Health Statistics (NCHS) implements NHANES. NCHS Ethics Review Board had approved the protocols, and subjects had signed the informed consent (13).

**Figure 1:**
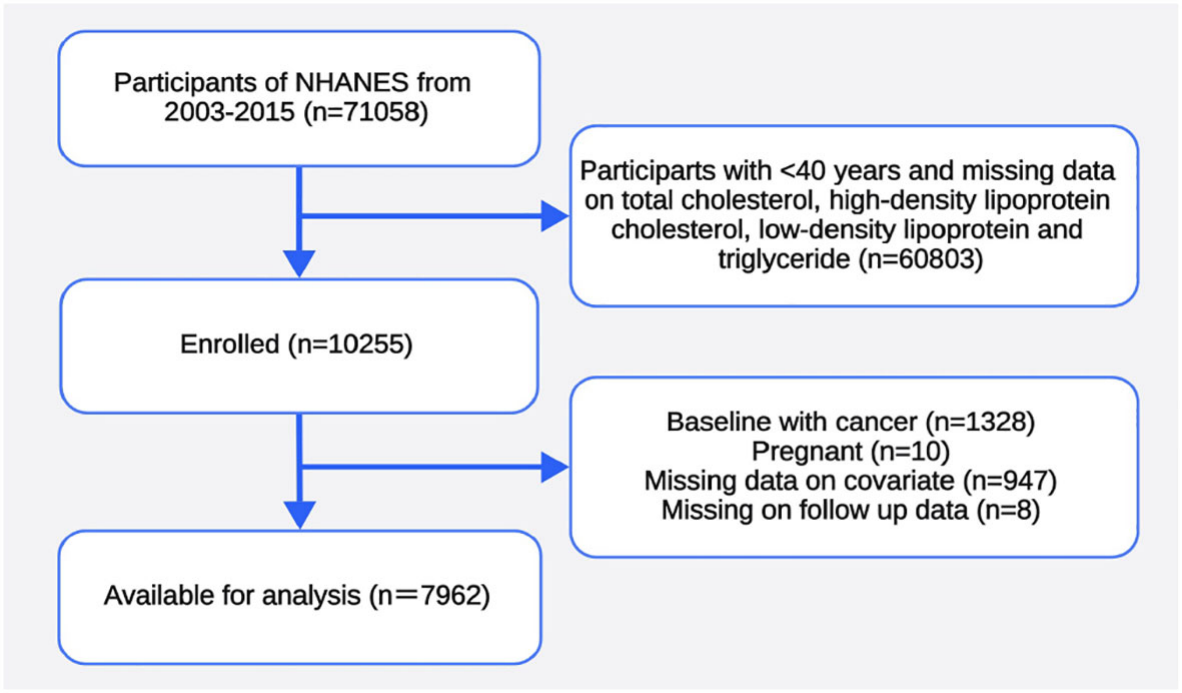
Flowchart of the sample selection process of the study population.

Data were extracted from the 2003–2015 cycles. We included participants with available data on TC, high-density lipoprotein cholesterol (HDL-C), low-density lipoprotein cholesterol (LDL-C), and triglycerides (TG). After excluding participants aged < 40 years (14), those with cancer at baseline, those with pregnant at baseline, those with incomplete covariate, and those with missing follow-up data, 7,962 individuals were enrolled for further analysis (**Figure 1**). All participants provided informed consent, and the program passed ethical review. Multiple imputation was used to replace missing values.

### 2.2 Study variables and covariates

RC was determined by subtracting TC from LDL-C and HDL-C. LDL-C levels were calculated from directly measured TC, TG, and HDL-C via the Friedewald equation, widely used in clinical practice and research settings (1, 3, 15). Enzymatic assays were used to measure TC in the laboratory. A direct immunoassay or heparin-manganese (Mn) precipitation method was used to measure HDL-C levels. The lipoprotein lipase technique was employed to quantify TG. The NHANES Laboratory/Medical Technologies Procedures Manual provided detailed instructions for collecting and processing specimens (12). The quality assurance and quality control (QA/QC) processes in the NHANES comply with the 1988 Clinical Laboratory Improvement Act requirements.

The study’s primary endpoint was all-cause mortality, and the population was followed for a mean of 109.2 (± 1.44) months. The primary endpoint is death from any cause during the follow-up, referred to as all-cause mortality. Professionals performed follow-ups. Secondary endpoints were non-cardiovascular and non-cerebrovascular death and cancer death.

Baseline demographic variables including age, gender, education levels (below high school, high school, above high school), race/ethnicity (Mexican, non-Hispanic White, non-Hispanic Black, other), poverty index ratio (< 1.3, 1.3 to 3.49, or ≥ 3.5) (16), marital status (married, alone), body weight, and height were collected from the household interview. Body mass index (BMI) was calculated as body weight divided by height squared (kg/m^2^) and classified participants into three weight-status groups: Normal (BMI < 25), overweight (BMI 25 to < 30), or obese (BMI ≥ 30) (17). The prevalence of the comorbidity (hypertension, diabetes) was recorded by a standardized medical condition questionnaire administered by trained interviewers. Information on smoking, alcohol use, medication (including lipid-lowering agents), and history of comorbidities had been obtained from the physical examination and associated questionnaire. Smoking habit was identified as someone who smoked 100 cigarettes in their lifetime. Alcohol user was defined as those who drank at least 12 alcoholic drinks in any one year. Blood pressure (BP) was calculated by averaging three consecutive BP readings after the participants rested calmly for five min. Hypertension was defined as systolic BP ≥140 mmHg and/or diastolic BP ≥ 90 mmHg, self-reported diabetes, or use of antihypertensive drugs (18). Diabetes was defined as fasting blood glucose ≥7.0 mmol/L, hemoglobin A1c (HbA1c) ≥ 6.5%, OGTT ≥ 11.0bmmol/L, or taking hypoglycemic drugs or self-reported diabetes (19). CKD-EPI Equation was used to calculate estimated glomerular filtration Rate (eGFR) (20), and the internationally recommended CKD staging based on eGFR level was used to assess the level of renal function of an individual (21). Individual liver function was assessed using The albumin-bilirubin (ALBI) grade (22). The albumin-bilirubin (ALBI) grade has emerged as an alternative, reproducible and objective measure of liver functional reserve in patients, defining worsening liver impairment across 3 grades (23).

The demographic data can be found in the demographics section of NHANES. Laboratory and questionnaire data can provide information on metabolic indicators and lifestyles. All the above variables are available for free at www.cdc.gov/nchs/nhanes/.

### 2.3 Statistical analysis

Weighted analysis was used as recommended by the NCHS, considering the oversampling of minorities to provide an accurate estimate of effects for the population because of NHANES’s complex multi-stage cluster survey design. Continuous variables were shown as population-weighted means with standard error (SE), while categorical variables were presented as frequency (percentage) (24).

The difference between the baseline characteristics was calculated using a population-weighted chi-square test (categorical variables) and a population-weighted linear regression model (continuous variables).

Cox regression hazard models were constructed to explore the association between the tertiary quantile RC levels (T1, T2, T3) and all-cause mortality, adjusted by several risk factors, including age, sex, race, smoking, education level, BMI, diabetes, hypertension, marital status, and alcohol consumption status, used to explore the potential non-linear relationship of RC with all-cause mortality among middle-aged and elderly and different subgroups.

Restricted cubic splines were used to explore the potential non-linear relationship of RC with all-cause mortality among US middle-aged and elderly and different subgroups. Multiple imputation was used to replace missing values.

Statistical analyses were performed via R version 4.3.2. P < 0.05 was regarded as statistically significant.

## 3 Results

### 3.1 Baseline characteristics

The weighted demographic features of the 7,962 subjects in the study grouped by RC are shown in **Table 1**, representing 104,991,870 U.S. people, for analysis and found associations between RC and mortality. The mean age was 56.30 ± 0.18 years. Of these subjects, 52.07% were men, and 72.43% were non-Hispanic white. The mean RC was 25.45 (± 0.24) mg/dl. During up to 109.2 (± 1.44) months of follow-up, 1,323 individuals died: 385 individuals died from cardiovascular disease, 290 from cancer, 80 from cerebrovascular disease, and 568 from other causes.

**Table 1:** Baseline characteristics of study population, NHANES, 2003–2015 (N=7,962).

Characteristics	Total (25.45 ± 0.24) n=7,962	T1 (13.48 ± 0.08) n=2,664	T2 (22.92 ± 0.08) n=2,651	T3 (41.88 ± 0.32) n=2,647	*P*-value
Age(year)	56.30 ± 0.18	55.58 ± 0.30	56.85 ± 0.30	56.56 ± 0.29	0.002
Sex, n(%)					< 0.001
Female	4,048(52.07)	1,291(51.74)	1,557(55.96)	1,200(47.90)	
Male	3,914(47.93)	1,265(48.26)	1,349(44.04)	1,300(52.10)	
TC (mg/dl)	56.30 ± 0.18	55.58 ± 0.30	56.85 ± 0.30	56.56 ± 0.29	0.002
TG (mg/dl)	199.71 ± 0.70	187.92 ± 0.93	200.35 ± 1.03	212.72 ± 1.14	< 0.001
LDL-C(mg/dl)	127.22 ± 1.22	67.44 ± 0.39	114.50 ± 0.40	209.35 ± 1.60	< 0.001
HDL-C(mg/dl)	118.71 ± 0.60	110.32 ± 0.80	122.64 ± 0.88	124.43 ± 1.01	< 0.001
Ethnic/Race, n (%)	55.55 ± 0.30	64.11 ± 0.49	54.79 ± 0.37	46.41 ± 0.35	< 0.001
Non-Hispanic White	3,636(72.43)	1,171(72.27)	1,233(70.06)	1,232(75.34)	
Non-Hispanic Black	1,603(10.74)	450(9.51)	892(16.42)	261(5.40)	
Mexican American	1,313(6.40)	453(7.03)	322(4.56)	538(7.89)	
Other	1,410(10.44)	482(11.19)	459(8.97)	469(11.37)	
BMI (kg/m^2)	29.32 ± 0.12	27.51 ± 0.17	29.62 ± 0.17	31.10 ± 0.20	< 0.001
BMI, n (%)					< 0.001
Normal	2,031(26.36)	620(24.38)	1,039(39.72)	372(12.89)	
Overweight	2,825(35.43)	927(36.45)	995(33.75)	903(36.35)	
Obese	3,106(38.21)	1,009(39.16)	872(26.52)	1,225(50.77)	
Education, n (%)					< 0.001
Below high school	1,099(6.76)	360(6.89)	308(5.52)	431(8.08)	
High school	2,234(25.12)	733(26.43)	731(21.72)	770(27.74)	
Above high school	4,629(68.12)	1,463(66.68)	1,867(72.76)	1,299(64.19)	
Smoke, n (%)					< 0.001
No	4,073(50.81)	1,332(51.31)	1,574(54.87)	1,167(45.61)	
Former	2,384(30.13)	759(30.72)	831(27.79)	794(32.26)	
Yes	1,505(19.05)	465(17.97)	501(17.34)	539(22.13)	
Diabetic, n (%)	2,047(19.62)	657(19.17)	567(13.97)	823(26.63)	< 0.001
Hypertensive, n (%)	4,303(48.67)	1,396(49.28)	1,430(41.63)	1,477(56.20)	< 0.001
Poverty ratio, n (%)					0.04
<1.3	2,319(17.80)	729(17.17)	789(17.04)	801(19.31)	
1.3–3.5	3,009(34.96)	989(35.96)	1,073(32.94)	947(36.28)	
>=3.5	2,634(47.24)	838(46.87)	1,044(50.01)	752(44.42)	
Alcohol, n (%)					0.004
Former	1,201(11.63)	395(11.98)	433(11.68)	373(11.21)	
Never	1,840(19.27)	588(19.03)	609(17.05)	643(22.09)	
Now	4,921(69.10)	1,573(68.99)	1,864(71.27)	1,484(66.71)	
Marital Status, n (%)					0.83
Alone	2,924(30.94)	917(30.68)	1,105(30.64)	902(31.55)	
Married	5,038(69.06)	1,639(69.32)	1,801(69.36)	1,598(68.45)	

Data are shown as n (weighted %), mean, or proportion (%). TC, total cholesterol; TG, Triglyceride; LDL-C, low‐density lipoprotein cholesterol; HDL-C, high‐density lipoprotein cholesterol; BMI, body mass index; NHANES, National Health and Nutrition Examination Survey.

Individuals with a higher RC tended to be older, male, frequent smokers, obese, diabetic, non-Hispanic White, hypertensive, less educated, and with a lower income.

### 3.2 Association of baseline RC with all-cause mortality

**Figure 2:**
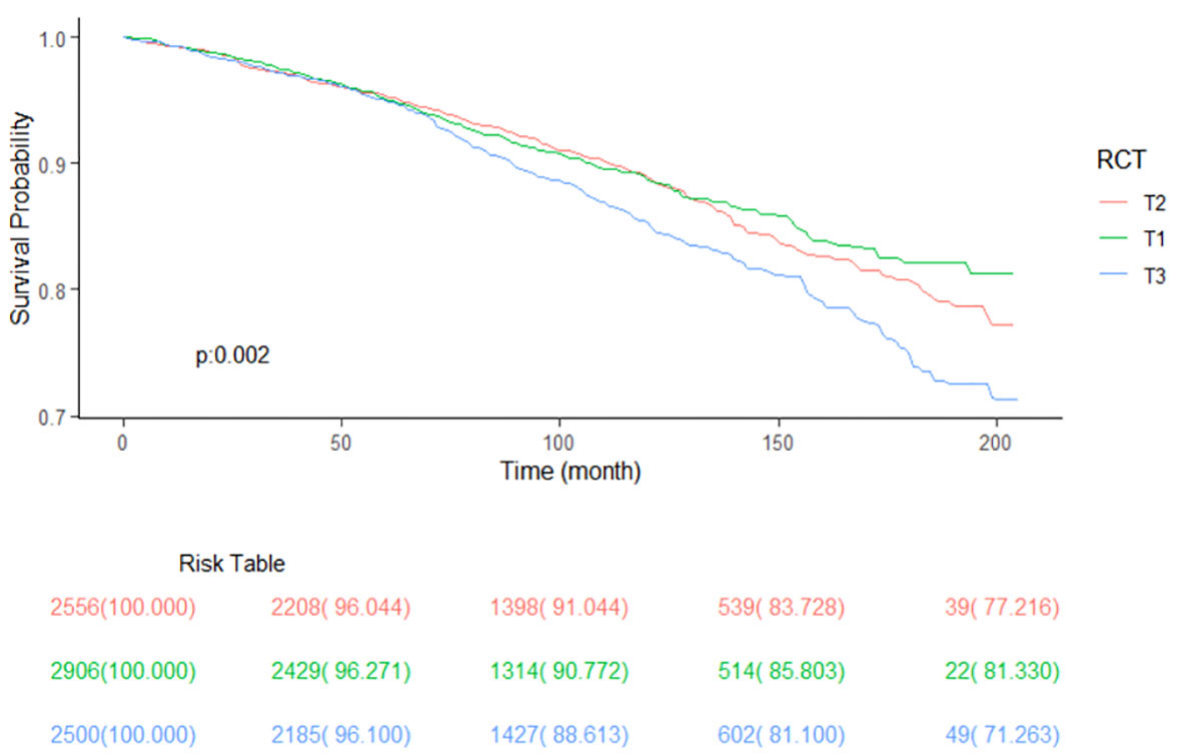
Kaplan-Meier survival curve, classified by RC level.

**Figure 3:**
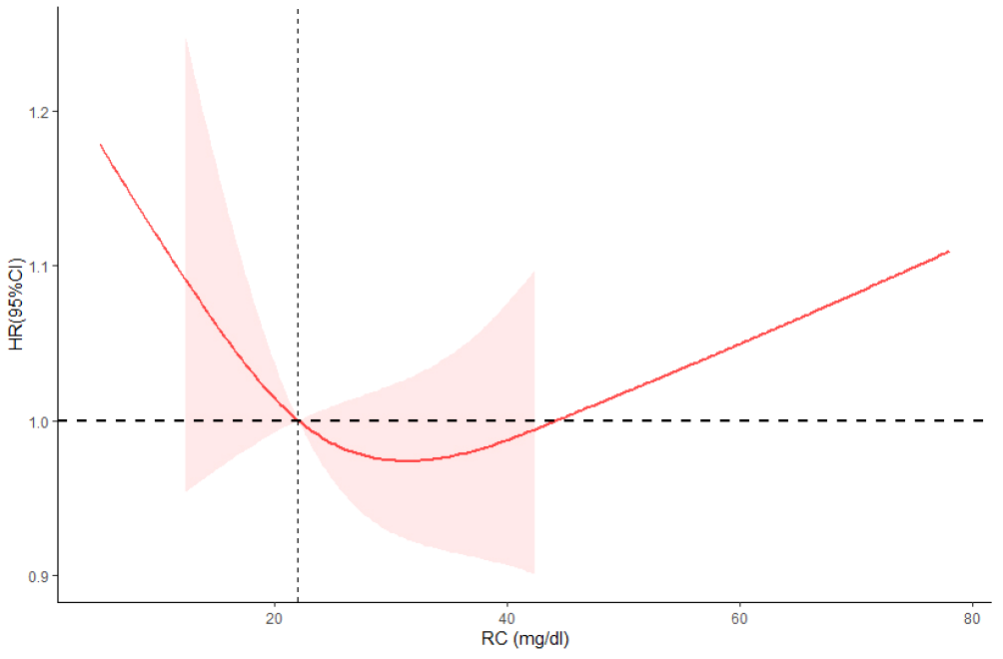
Restricted cubic spline plots between RC and all-cause mortality. Analysis was adjusted for Age + Sex + Race/Ethnic + Smoking + Hypertension + Diabetes + CKD + ALBI_Grade + Alcohol + Poverty + Marital Status + BMI. HR, hazard ratio; CI, confidence interval. Non-linear-*P* = 0.21.

As we can see from **Table 2**, T2 has no significant association with T1 (0.95 [0.80, 1.13], *P* = 0.57) but has significantly lower all-cause mortality than T3 (1.27 [1.09, 1.49], *P* = 0.003) in the crude model. After adjusting for sex, age, and race (model 2), T2 has no significant association with T1 (1.10 [0.94, 1.28], *P* = 0.22) but has significantly lower all-cause mortality than T3 (1.35 [1.19, 1.54], *P* < 0.001). Model 3 adjusted for marital status, BMI, education level, smoking, alcohol consumption, diabetes, CKD, ALBI_Grade and hypertension based on model 2. T2 has significantly lower all-cause mortality than T1 (1.19 [1.02, 1.39], *P* = 0.02) and T3 (1.24 [1.07, 1.43], *P* = 0.004). **Figure 2** shows the different levels of RC groups of KM survival curve. **Figure 3** shows the relationship between RC and all-cause mortality.

**Table 2:** Association of baseline RC with all-cause mortality.

	model1		model2		model3	
RC	HR (95% CI)	*P*-value	HR (95% CI)	*P*-value	HR (95% CI)	*P*-value
T1	0.95(0.80,1.13)	0.57	1.10(0.94,1.28)	0.22	1.19(1.02, 1.39)	**0.02**
T2	ref	ref	ref	ref	ref	ref
T3	1.27(1.09,1.49)	**0.003**	1.35(1.19,1.54)	**<0.001**	1.24(1.07, 1.43)	**0.004**

HR, hazard ratio; CI, confidence interval. model 1: crude model. model 2: adjusted for Age + Sex + Race. model 3: model 2 + BMI + Education + Smoke + Diabetes + CKD + ALBI Grade+ Hypertension+ Alcohol +Married Status + Poverty index ratio. P < 0.05 was regarded as statistically significant.

### 3.3 Subgroup analysis

**Figure 4:**
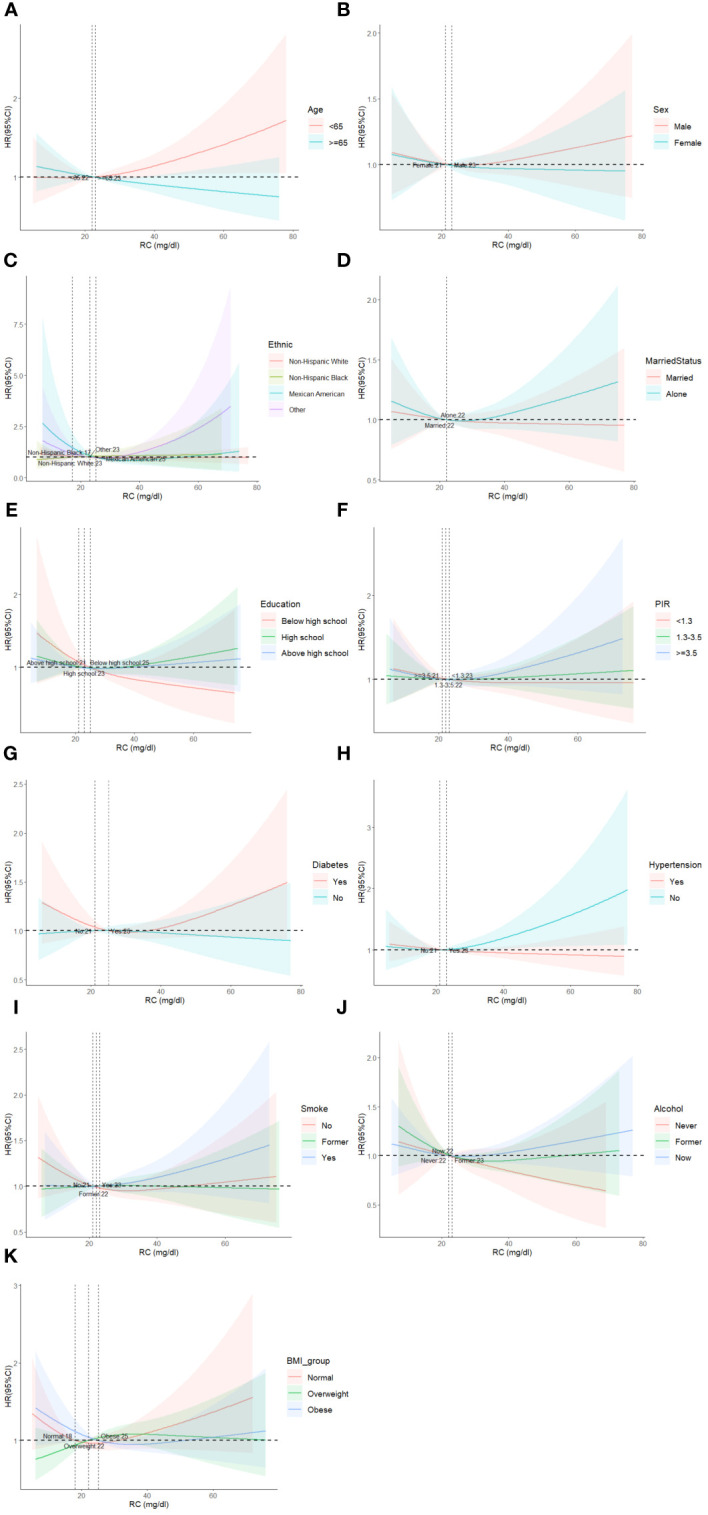
Restricted cubic spline plots of the association between RC and cardiovascular mortality in subgroups. Analyses by Age **(A)**, Sex **(B)**, Race/Ethnic **(C)**, Marital Status **(D)**, Education **(E)**, Poverty ratio index **(F)**, Diabetes **(G)**, Hypertension **(H)**, Smoke **(I)**, Alcohol **(J)** and BMI **(K)**. Analysis was adjusted for Age + Sex + Race + Smoking + Hypertension + Diabetes + CKD + ALBI_Grade + Alcohol + Poverty + Marital Status + BMI. HR, hazard ratio; CI, confidence interval.

**Figure 5:**
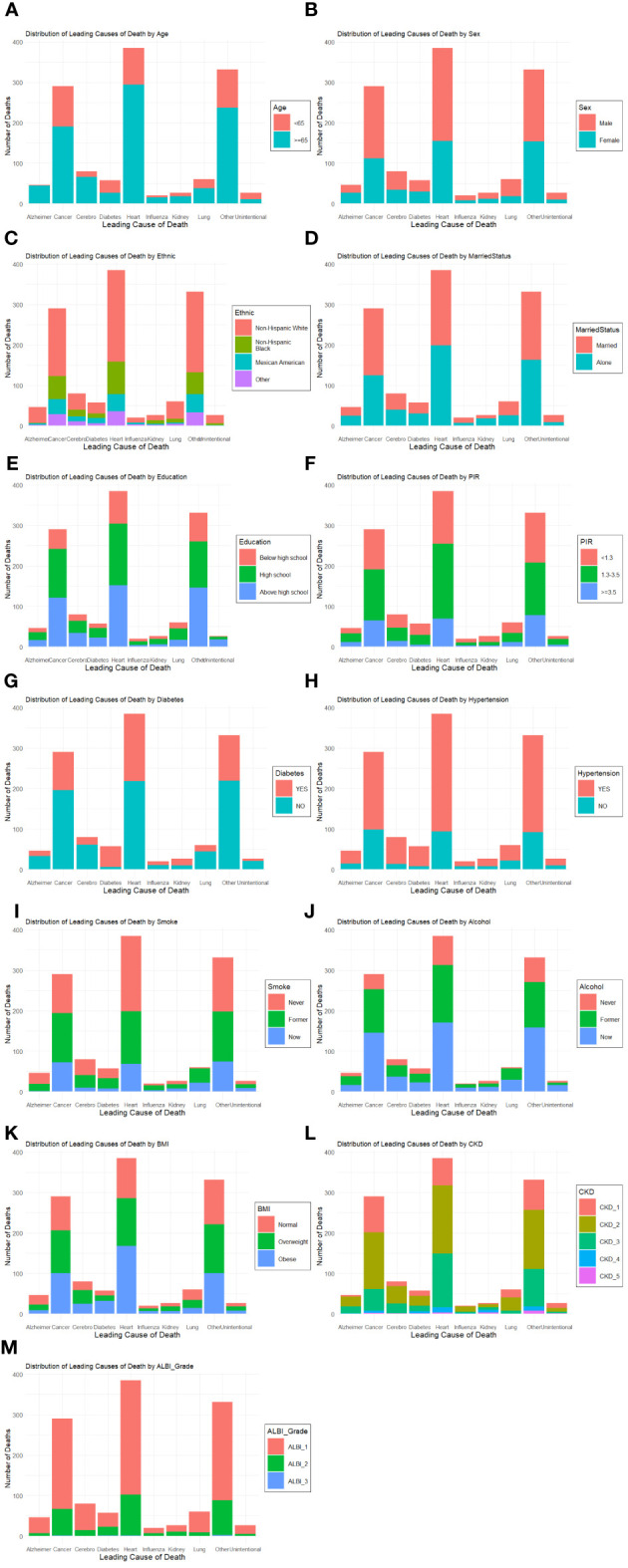
Plots of the proportion of deaths in each subgroup. Analyses by Age **(A)**, Sex **(B)**, Race/Ethnic **(C)**, Marital Status **(D)**, Education **(E)**, Poverty ratio index **(F)**, Diabetes **(G)**, Hypertension **(H)**, Smoke **(I)**, Alcohol **(J)**, BMI **(K)**, CKD **(L)** and ALBI_Group **(M)**.

In subgroup analysis, there was no significant difference in the association between RC levels and all-cause mortality among different subgroups (**Figure 4**). Based on the causes of death of different subgroups and populations, we plotted the proportion of causes of death for each subgroup (**Figure 5**).

### 3.4 RC level and cause of death

#### 3.4.1 Cardiovascular death

RC in all models was not significantly associated with cardiovascular death. In model 1, T2 exhibits no significant association with T1 (0.82 [0.28, 1.18], *P* = 0.28), while its association with T3 (1.09 [0.82, 1.45], *P* = 0.55) is not statistically significant. In model 2, T2’s association with T1 (0.95 [0.65, 1.37], *P* = 0.77) and T3 (1.19 [0.90, 1.57], *P* = 0.22) indicates no significant association. Finally, in model 3, T2 correlates with T1 (1.10 [0.76, 1.60], *P* = 0.62), while its association with T3 (1.06 [0.79, 1.42], *P* = 0.69) remains no statistically insignificant.

#### 3.4.2 Non-cardiovascular and non-cerebrovascular death

**Table 3** demonstrates the association between RC levels and non-cardiovascular and non-cerebrovascular death. Models are adjusted in the same as in **Table 2**. In model 1, T2 exhibits a significant association with T1 (1.06 [0.86, 1.30], *P* = 0.58), while its association with T3 (1.40 [1.16, 1.68], *P* < 0.001) is statistically significant. In model 2, T2’s association with T1 (1.22 [1.01, 1.48], *P* = 0.04) and T3 (1.46 [1.25, 1.70], *P* < 0.001) indicates a significant association. Finally, in model 3, T2 significantly correlates with T1 (1.29 [1.06, 1.55], *P* = 0.01), while its association with T3 (1.34 [1.13, 1.58], *P* < 0.001) remains statistically insignificant.

**Table 3:** Cox models for different death for RC.

	Cardiovascular Death					
	model1		model2		model3	
	HR (95% CI)	*P*-value	HR (95% CI)	*P*-value	HR (95% CI)	*P*-value
T1	0.82(0.57,1.18)	0.28	0.95(0.65,1.37)	0.77	1.12(0.76,1.65)	0.57
T2	ref	ref	ref	ref	ref	ref
T3	1.09(0.82,1.45)	0.55	1.19(0.90,1.57)	0.22	1.03(0.77,1.38)	0.86
RC	1.01(1.00,1.02)	0.11	1.01(1.00,1.02)	0.13	1.00(0.99,1.01)	0.93

	Non-cardiovascular and Non-cerebrovascular Death					
	model1		model2		model3	
	HR (95% CI)	*P*-value	HR (95% CI)	*P*-value	HR (95% CI)	*P*-value
T1	1.06(0.86,1.30)	0.58	1.22(1.01,1.48)	**0.04**	1.29(1.06,1.55)	**0.01**
T2	ref	ref	ref	ref	ref	ref
T3	1.40(1.16,1.68)	**<0.001**	1.46(1.25,1.70)	**<0.001**	1.34(1.13,1.58)	**<0.001**
RC	1.01(1.00,1.01)	0.07	1.01(1.00,1.01)	0.13	1.00(0.99,1.01)	0.93

	Cancer Death					
	model1		model2		model3	
	HR (95% CI)	*P*-value	HR (95% CI)	*P*-value	HR (95% CI)	*P*-value
T1	1.27(0.87,1.84)	0.21	1.42(0.98,2.06)	0.06	1.48(1.01,2.15)	0.06
T2	ref	ref	ref	ref	ref	ref
T3	1.81(1.33,2.47)	**<0.001**	1.89(1.43,2.51)	**<0.001**	1.83(1.38,2.43)	**<0.001**
RC	1.01(1.00,1.02)	**0.04**	1.01(1.00,1.02)	0.05	1.01(1.00,1.02)	0.15

P < 0.05 was regarded as statistically significant.

#### 3.4.5 Cancer death

**Table 3** shows a significant link between RC levels and cancer death. Models are adjusted in the same as in **Table 2**. In model 1, T2 has no significant association with T1 (1.27[0.87, 1.84], *P* = 0.21) and T3 (1.81 [1.33, 2.47], *P* < 0.001), indicating a significant association. In model 2, T2 has no significant association with T1 (1.42 [0.98, 2.06], *P* = 0.06) but has significantly higher cancer mortality than T3 (1.89 [1.43, 2.51], *P* < 0.001). In model 3, T2 has significantly lower cancer mortality than T1 (1.43 [0.99, 2.08], *P* = 0.06 and T3 (1.83 [1.38, 2.43], *P <* 0.001). **Figure 5** shows the KM survival curve with significant differences in cancer survival rates and non-cardiovascular and non-cerebrovascular survival between RC levels (*P* = 0.004). The T2 group had a higher cancer survival probability than T1 and T3 groups (*P* = 0.001).

**Figure 6:**
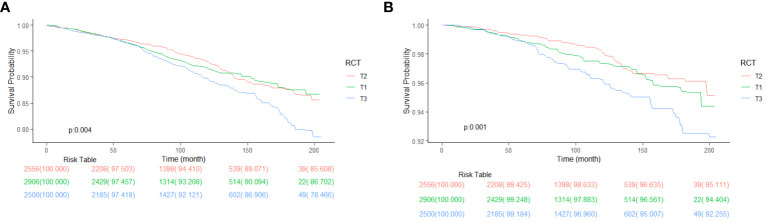
Kaplan-Meier survival curve between Non-cardiovascular and Non-cerebrovascular Survival **(A)**, Cancer Survival **(B)** and RC, classified by RC levels.

**Figure 7:**
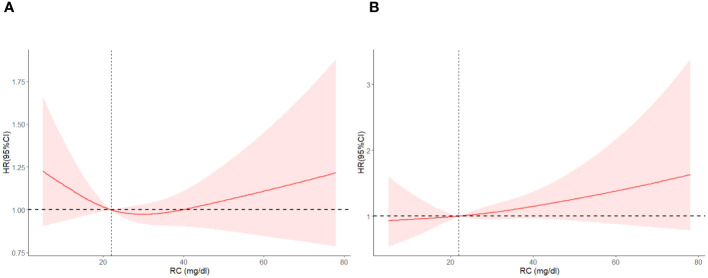
Restricted cubic spline plots between Non-cardiovascular and Non-cerebrovascular Death **(A)** and Cancer Death **(B)** and RC.

**Figure 6** shows the Restricted cubic spline plots between non-cardiovascular and non-cerebrovascular survival (A), cancer survival (B), and RC. In **Figure 6**, the Non-linear-*P* for non-cardiovascular and non-cerebrovascular death (A) was 0.09, the Non-linear-*P* for cancer death (B) was 0.67. **Figure 7** shows the restricted cubic spline plots between Non-cardiovascular and Non-cerebrovascular Death (A) and Cancer Death (B) and RC.

## 4 Discussion

### 4.1 Analysis

Our study included 7,962 middle-aged and older adults, representing 104,991,870 U.S. people, and found associations between RC and mortality. Firstly, we discovered that the lower RC group T1 (1.20 [1.02, 1.40], *P* = 0.03) and the higher RC group T3 (1.21[1.05,1.40], *P* = 0.01) are both associated with a higher risk of all-cause mortality. Secondly, RC levels are not associated with cardiovascular death. Moreover, the lower RC level T1 is associated with higher non-cardiovascular and non-cerebrovascular death (1.25[1.13,1.38], *P*<0.001). Furthermore, lower and higher RC levels are associated with higher cancer deaths. The lower group T1 (1.47 [1.01, 2.15], *P* = 0.04), the higher group T3 (1.80 [1.36, 2.38], *P* < 0.001).

The study shows that older, male, non-Hispanic white, obese, less educated, frequent smokers, diabetic, hypertensive, low income, and never-drink-alcohol people tend to have higher RC. Similar to our findings, the Castaner study showed that people with obesity and hypertension are more likely to have higher levels of RC (4); Hu et al. found that people with low education and low income had higher RC levels (9). Tian et al. found that people with hypertension and diabetes had a higher level of RC (25). Interestingly, the study showed that people who never drank alcohol people are more likely to have higher RC. That might be because of moderate alcohol consumption. Although, there was no confirmed evidence of protection from moderate drinking for all-cause and cause-specific mortality (26, 27).

We found a U-shaped curve between RC and mortality; all-cause mortality was lowest in the middle tertile (T2) of RC and increased in the lowest (T1) or highest tertile (T3). A cohort study, which included approximately 90,000 Danish general population, showed lower all-cause mortality in people with intermediate RC levels, similar to our results (15). Tian et al. included 3,403,414 community-based participants from ChinaHEART, an ongoing government-funded public health program throughout China. They found that the second percentile levels of RC concentrations were associated with the lowest all-cause mortality (28).

A study included 2823 patients with heart failure shows that low remnant cholesterol levels are associated with increased all-cause mortality in HF patients. And speculated that high cholesterol levels and higher CRP level can improve myocardial energy level and ability to resist infection in patients with heart failure, thus improved the prognosis of patients (15). But there is no clear research and clinical evidence of this association, their relationship still needs further research. A cohort study including 5,414 Danish patients diagnosed with ischemic heart disease found that the risk of all-cause mortality increased with higher RC concentrations (29). Horace et al. also found that increasing RC levels increased the risk of all-cause mortality (30). A Mendelian randomization design genetically determined that increased remnant cholesterol concentrations were associated with increased IHD risk (4, 31). Furthermore, genetically increased RC was also associated with low-grade inflammation (3). This mechanism might explain how RC contributes to increased all-cause mortality. A systematic review including 30,605 patients suggests that elevated concentration RC may independently predict MACEs in patients with CHD (32). Our study showed that the middle but not the lowest RC levels were associated with the lowest all-cause mortality in middle-aged and older adults. There might be extreme concentrations of HDL-C or LDL-C, resulting in higher all-cause mortality (14, 33, 34). RC levels are inversely associated with HDL-C and LDL-C. The findings suggested that people may benefit from the middle RC levels ranging from 20–30 mg/dl.

In our study, cardiovascular mortality was not statistically associated with RC level. On the contrary, a contemporary population-based cohort including 87,192 individuals from the Copenhagen General Population Study aged 20–69 years found that elevated RC is associated with increased risk of mortality from cardiovascular and other causes but not from cancer (35). Differences in results may be due to differences in the included populations and the covariates, which we additionally adjusted for hypertension. Additionally, LDL-C, which is inversely associated with RC, is more strongly associated with cardiovascular outcomes (34). LDL-C levels are the target of lipid-lowering for ASCVD prevention and treatment (36).

These findings suggest that extreme RC levels were associated with higher cancer mortality. Similarly, an analysis of the US population showed higher cancer mortality all-cause mortality at higher RC (37). Observational studies have shown that lower HDL-C levels are associated with higher cancer mortality (34), and lower HDL-C may cause higher cancer mortality at higher RC levels. On the contrary, a Chinese cohort suggested that higher levels of RC were associated with lower cancer mortality (25). Because cholesterol is essential in regulating immune cell function, high serum cholesterol levels enhance the antitumor function of natural killer cells (38). There may be a weaker inhibitory effect on the tumor at low RC levels. A recent analysis of residual cholesterol and cancer death in a limited Nordic population was negative (35). The association and mechanism between RC level and cancer mortality have not been concluded, and further study is needed.

The study indicated that lower RC levels were significantly associated with higher non-cardiovascular and non-cerebrovascular mortality. However, the association with increased mortality from other causes is complex to explain since the relationship between elevated RC, plasma triglycerides, and most non-cardiovascular diseases is unclear (35).

We demonstrated stability between RC and mortality across population subgroups. The results were similar across multiple subgroup analyses, indicating that the association between RC and all-cause mortality was consistent across gender, age, race, health status, and social relationships. Tian et al. (25) and Wadstrom et al. (35). obtained similar results in the subgroup analysis, with no significant differences in age, gender, smoking, diabetes, and BMI levels between the different groups.

### 4.2 Strengths and limitations

The strength of this study is the use of a large sample size, long-term follow-up cohort data, and comprehensive consideration and adjustment of the risk factors involved. Also, sampling weights developed by the NCHS to minimize nonresponse bias were used in our analyses; we used weights, and the data better represent the actual population. Lipid profile levels were from standard laboratory measurements, which makes the results easily translatable to real-world clinical settings. Furthermore, the subgroup analyses were consistent, indicating stable results. Besides, the comprehensive collection of baseline information made extensive adjustment and stratification possible, which clarifies and strengthens the results. Most importantly, this study showed a U-shaped association between RC and all-cause mortality, suggesting we should focus on lowering LDL cholesterol concentrations and keeping RC at moderate levels.

Our study has several limitations. Firstly, as an observational study, it is prone to confounding and cannot conclude causality. Given the variability of RC concentrations, the use of a single measurement of a patient’s lipid profile at study enrollment without repeated sampling will lead to regression dilution bias; thus, the present results should be viewed as minimal estimates, whereas the actual risk increase in all-cause mortality for increased remnant cholesterol likely is higher than that observed in the present study. Thus, future studies are needed to focus on the incidence of all-cause mortality and causes of death based on Mendelian randomization or other methods to address causal questions better.

## 5 Conclusion

In conclusion, both lower or higher RC levels were associated with higher mortality. These results suggest that individuals with RC at the extremes may have a higher mortality risk and may require more attention. Maintaining RC may be a target for regulating cholesterol to reduce mortality risk. Our study highlights the predictive value and uniqueness of RC in estimating all-cause mortality. By recognizing the importance of RC, clinicians can improve risk stratification, enhance patient management, and ultimately improve patient outcomes.

## Data availability statement

The original contributions presented in the study are included in the article/supplementary material. Further inquiries can be directed to the corresponding authors.

## Ethics statement

The studies involving humans were approved by National Center for Health Statistics Institutional Review Board of the United States. The studies were conducted in accordance with the local legislation and institutional requirements. The participants provided their written informed consent to participate in this study.

## Author contributions

MB: Writing – original draft, Data curation, Formal analysis, Software. JL: Data curation, Formal analysis, Writing – original draft. YW: Writing – original draft, Visualization. ML: Visualization, Writing – original draft. CW: Supervision, Writing – review & editing. JZ: Writing – review & editing, Supervision. MS: Supervision, Writing – review & editing.
